# Increase in Auxeticity Due to the Presence of a Disordered Crystalline Phase of Hard Dumbbells Within the Nanolayer–Nanochannel Inclusion Introduced to the f.c.c. Hard Sphere Crystal

**DOI:** 10.3390/ma17225558

**Published:** 2024-11-14

**Authors:** Jakub W. Narojczyk

**Affiliations:** Institute of Molecular Physics, Polish Academy of Sciences, M. Smoluchowskiego 17, 60-179 Poznań, Poland; narojczyk@ifmpan.poznan.pl

**Keywords:** auxetics, negative Poisson’s ratio, nanolayers, hard spheres, inclusions, Monte Carlo simulations

## Abstract

To obtain materials or metamaterials with desired elastic properties that are tailor-made for a particular application, it is necessary to design a new material or composite (which may be cumbersome) or to modify the structure of existing materials in order to change their properties in the desired direction. The latter approach, although also not easy, seems favourable with respect to parameters like costs and time-to-market. Despite the fact that elastic properties are one of the oldest studied physical parameters of matter, our understanding of the processes at the microstructural level, that are behind these properties, is still far from being complete. The present work, with the help of Monte Carlo computer simulations, aims to broaden this knowledge. The previously studied model crystal of hard spheres, containing a combined nanolayer and nanochannel inclusions, is revisited. This periodic model crystal has been extended to include a degree of disorder in the form of degenerate crystalline phase by introducing a degenerate crystalline phase within its structure. The inclusion has been transformed (without changes to its shape, size, or orientation) by randomly connecting the neighbouring spheres into di-atomic molecules (dumbbells). The impact of this modification on elastic properties has been investigated with the help of the Parrinello–Rahman approach in the isothermal–isobaric ensemble (NpT). It has been shown, that the presence of the degenerate crystalline phase of hard dumbbells in the system leads to a significant decrease in the Poisson’s ratio in [110]-direction (ν=−0.235) and an overall enhancement of the auxetic properties.

## 1. Introduction

The elastic properties are one of the earliest studied characteristics of materials. To date, most of these properties are very well measured and documented for most materials that are used. This, however, does not help us when facing a problem that requires a material with properties that go beyond what can be achieved for conventional ones. The demand for materials with new or specific properties is proportional to the increasing number of possible applications. It was not long ago when the materials exhibiting negative Poisson’s ratio [1] (although theoretically possible) were thought to be non-existent. This was due to the fact that the ability of some natural materials to exhibit negative Poisson’s ratio in only some of the directions, e.g., most of the cubic crystalls [2], has not yet been noticed. At present, *auxetics* [3], as they came to be called, are a well established class of materials that are undergoing constant development [4] by scientists and engineers. It has been almost 40 years since the pioneering works by Lakes [5] (who fabricated the first man made auxetic foam) and Wojciechowski [6,7,8], who formulated and solved the first thermodynamically stable model showing and explaining the nature of these extraordinary properties. Despite the significant effort in the study of auxetics, their practical applications, to date, are rare. However, a positive sign is their increase in recent years. The most noteworthy are the attempts to apply auxetic materials and structures in medical, bioengineering, and personal protection areas, as they have the chance to directly impact human health and wellbeing. In particular, the creation of auxetic micropatterned cardiac patches, obtained by excimer laser microablation, with the mechanical properties of the patches matching those of the native heart of the patients’ tissue, were reported by Stevens et al. [9]. A recent report by Kaufman et al. [10] presented an animal study, where modified stents that incorporated an auxetic structure were implanted to adult ovine. Their findings have shown that auxetic stents immediately increased the vein inflow area. Graziosi et al. investigated the feasibility of using auxetic patterns to enhance kinesiology tapes [11] used in the treatment of musculoskeletal injuries that are frequent among young athletes and physically active adults. Sport is another area where the auxetic materials are utilized in the form of enhanced personal protection apparel end equipment [12,13]. The design and fabrication of such personal apparel will also require enhanced fabrics. A recent review by Chang and Hu [14] discussed auxetic knitted, woven, and non-woven fabrics. Applications of auxetics in engineering sectors can also be found, e.g., in furniture design, where the use of auxetic springs [15] and dowels [16] has been investigated. The auxetic materials have been studied both theoretically and experimentally [17,18,19,20,21]. The latter focused on the development and fabrication of (among others) negative Poisson’s ratio polymers [22,23], composites [24,25], foams [26,27,28], fabrics [29,30,31,32], and metamaterials [33]. Alderson et al. first showed that it is possible to produce auxetic polypropylene fibres on an industrial, large-scale extruder [22] and produced fibres with a high degree of auxeticity. Novak et al. fabricated a chiral auxetic sandwich panel composite [24] and performed ballistic testing, both experimentally and numerically, to investigate the impact resistance of such a structure. Dalcanale et al. [25] proposed a promising solution to the problem of generating artificial molecular auxetics. The proposed auxetic polymer did not mimic metamaterials at the molecular level. Rather, it relied on the approach of mechanically driven conformational expansion. The matrix of the proposed polymer of intrinsic microporosity presents a rigid structure that is capable of transferring the mechanical stress into a conformational expansion of the crosslinking units. On the other hand, the theoretical studies on auxetic materials focus mainly on the development of new models that show a negative Poisson’s ratio [34,35,36,37,38,39,40,41,42,43,44,45,46,47,48], which are later solved with a variety of computer simulation techniques, from which Finite Element Method [42,49,50,51,52] for macroscopic models and Monte Carlo [53,54,55,56] or molecular dynamics [57] for atomistic models, are the two most popular approaches. The development of auxetic materials can be roughly divided into two categories: the design of new materials or models with auxetic properties, and the study of available materials or models in order to enhance their elastic properties. The atomic models are especially interesting as they relatively easily allow testing various modifications to materials’ structures and assess their impact on macroscopic elastic properties. In natural volumetric systems, such as liquid crystals, characterized by a regular periodic arrangement of atoms or molecules that exhibit isotropic or anisotropic properties dependent on the alignment, the inclusions and/or defects, can significantly affect there elasticity and viscosity [58,59]. It has been shown that modifying the crystalline structure using inclusions to introduce different types of particles into a material, one can significantly change its elastic properties [60,61]. However, depending on the size, shape, or orientation of the inclusions, the result may be either enhancement [60] or removal [61] of auxetic properties. This shows that we need to gain a deeper understanding on how the microstructural modifications impact macroscopic elastic properties (e.g., Poisson’s ratio).

In this work, the impact of the disorder introduced into an otherwise periodic crystalline system will affect its Poisson’s ratio. Disorder is an important aspect as, in reality, we always deal with a certain level of defects or particle size dispersion [54,62,63,64], and their impact on the elastic properties has to be studied and taken into consideration in practical applications. Disorder may be considered in a variety of aspects. In 1987, Wojciechowski theoretically predicted [65] the existence of an aperiodic solid phase in the two-dimensional (2D) system of hard dumbbells when they are close to dimers, i.e., when the distance between the centres of the discs forming a dumbbell is close to the diameter of the discs. This phase was initially coined by him as the disordered crystal [65] and, finally, the *degenerate crystal* (DC) [66,67,68]. That prediction was soon confirmed by Monte Carlo simulations of systems of 2D dimers [66,67,69] and dumbbells [70]. The existence of the DC phase was also confirmed for three-dimensional dimers [71]. The characteristic feature of a degenerate crystal is the lack of periodicity in positions and orientations of molecules, where, at the same time, the positions of atoms that form the molecules are periodic and can form, e.g., a hexagonal 2D lattice or f.c.c. and h.c.p. in 3D. The use of this phase was chosen for this study because it can be easily applied to existing models with studied previously inclusions [61] and its impact on elastic properties when used with additional complex inclusion sets have not been investigated. As mentioned in the previous paragraph, the introduction of combined nanolayer and nanochannel inclusions into the hard sphere f.c.c. crystal resulted in an increase in Poisson’s ratio values and the removal of the auxetic properties. The objective of this work is to introduce a disordered crystalline phase into such a model and to investigate its impact on the elastic properties of such system.

This article has the following structure. In Section 2, the studied models are described. The research method is briefly reviewed in Section 3. The discussion of the results is conducted in Section 4. Section 5 contains the summary and conclusions.

## 2. The Model

**Figure 1:**
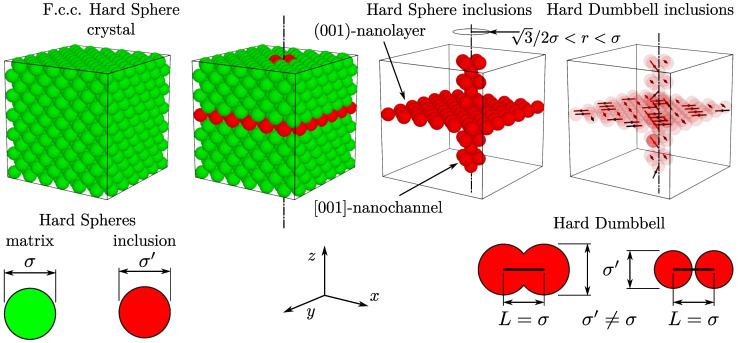
Visualization of the studied models. The f.c.c. HS crystal with the [001]-nanochannel and (001)-nanolayer inclusions filled by either hard spheres or hard dumbbells. In all cases, inclusion spheres have the diameter $\sigma^{\prime }$, that is different from the diameter of matrix spheres (σ).

The model studied in this work is an extension of the model studied in [61]. It consists of *N* hard spheres with a diameter σ, initially forming the f.c.c. lattice at close packing. The value σ is also treated as the unit of length. For such a system, inclusions are introduced by replacing some of the particles with other hard spheres of a different diameter ($\sigma^{\prime }$). The choice of the shape and size of the inclusions is arbitrary; here, only the combination of a single nanochannel and nanolayer is considered. The nanochannel is oriented in the [001]-direction and has a radius equal to σ. All spheres forming the original system (which will be further referenced as the *matrix crystal*), whose centres of mass are positioned at close packing within this distance from a selected crystalline axis, are replaced with the $\sigma^{\prime }$-spheres. The nanochannel radius has been selected, such that only the particles lying directly on the crystalline axis and their nearest neighbours are included. The nanolayer is oriented orthogonally to the nanochannel, and, similarly, all spheres that form a selected crystallographic plane are replaced with inclusion spheres. This is illustrated in Figure 1. Such a case, where the inclusion is formed by hard spheres, has been studied in [61]. Here, an additional condition (constraint) is introduced into the model by randomly paring the neighbouring inclusion spheres into diatomic molecules—hard dumbbells. Thus, the degenerate crystal phase is obtained within the inclusion. The elastic properties of a 3D degenerate crystalline system with nanochannel inclusions filled with hard spheres has been studied in [72].

The interatomic interactions in this work are modelled with the hard sphere (HS) potential of the form:

(1) $$\beta u_{ij}=\left\{\begin{array}{cc} \infty , & r_{ij}<\sigma_{ij},\\ 0, & r_{ij}\geq \sigma_{ij}, \end{array}\right.$$

where $r_{ij}$ is the distance between the centres of spheres *i* and *j* and $\sigma_{ij}$ stand for the sum of the radii of these spheres, $\beta =1/(k_{B}T)$, $k_{B}$ is the Boltzmann constant, and *T* is the temperature. This simple, athermal and purely geometric interaction is a fundamental reference model in the theory of liquids and condensed matter physics [73,74], and is also extremely useful in the study of elastic properties, as hard models can exhibit a negative Poisson’s ratio. As mentioned, this geometrical interaction is only sensitive to the change in dimensions and shape of interacting particles. Thus, the inclusion spheres have the diameters $\sigma^{\prime }\neq \sigma$. The impact of the inclusions will be analyzed with respect to the ratio $\sigma^{\prime }/\sigma$, which is the ratio of the inclusion to the matrix sphere diameters.

It is important to note that the described model is considered in the periodic boundary conditions. The studied supercell, formed by selected *N* particles, is surrounded with the 26 identical copies of itself. Thus, one obtains a system with periodically stacked nanolayers combined with a periodic array of nanochannels. Visualization of periodic boundaries is given in [61] (see Figure 1 therein).

## 3. The Method

### 3.1. Elastic Properties

The Parrinello–Rahman [75,76,77] idea has been used with the Monte Carlo simulation method to determine the elastic properties of the model described in the previous section. Similar to [61], modelled supercells have been studied in the isobaric–isothermal ensemble (NpT), i.e., the number of particles, external hydrostatic pressure and temperature were kept constant during the simulation. To briefly summarize, the shape fluctuations [75,76,77] of the parallelepiped volume that contained the studied system, and described by a symmetric matrix h (formed by vectors defining its edges), allow for determining the elastic strain tensor ε:

(2) $$\begin{array}{c} \varepsilon =\frac{1}{2}\left(h_{p}^{-1}.h.h.h_{p}^{-1}-I\right), \end{array}$$

where I is a unit matrix. The reference matrix $h_{p}$ is equivalent to 〈h〉, i.e., the average value of the **h** matrix under the dimensionless pressure $p^{*}=p\beta \sigma^{3}$, where $\beta =1/k_{B}/T$, *T* is the temperature, and $k_{B}$ is the Boltzmann constant. The strain tensor elements can be used to calculate the complete elastic compliance tensor S [61,77], a fourth rank symmetric tensor of 81 components in the case of the lowest symmetry. The knowledge of S allows one to fully describe the elastic properties of any crystal. It can be used to calculate the Poisson’s ratio in any crystallographic direction using the form [78,79]:

(3) $$\nu_{nm}=-\frac{m_{\alpha }m_{\beta }S_{\alpha \beta \gamma \delta }n_{\gamma }n_{\delta }}{n_{\zeta }n_{\eta }S_{\zeta \eta \kappa \lambda }n_{\kappa }n_{\lambda }}\;.$$

$\vec{n}$ and $\vec{m}$ are the pair of unit vectors in a mutually orthogonal direction in which, respectively, the external load is applied to the system and in which Poisson’s ratio is measured. In Equation (3), indices *n* and *m* correspond to these vectors, where $n_{\alpha }$ and $m_{\beta }$ are their respective components. It should be noted that the Einstein summation convention has been used on Greek indices. Moreover, for clarity, the S tensor has been replaced with the elastic compliance matrix S (a symmetric square matrix of dimension six) using the Voigt representation [80]. The Latin indices for $S_{ij}$ elements are equal to i,j=1,…,6. For further details on the method used here to calculate elastic properties of the studied models, please see [61,77].

### 3.2. Computation Details

The selected size of the sample used for simulations was N=864 spheres, which corresponds to 6×6×6 f.c.c. unit cells. The combined nanochannel and nanolayer inclusions consisted of $N_{\mathrm{inc}}=101$ (11.68% of *N*). The selected size has been previously verified to provide accurate results, by simulating systems doubled in all three directions [81]. To avoid the diffusion of particles from the inclusion to the matrix and to facilitate a comparison of the results with the ones reported in [61], the external hydrostatic (dimensionless) pressure has been set to $p^{*}=100$. It is worth adding that this is only one order of magnitude higher than the pressure under which the hard sphere system undergoes melting. Different values of pressure have also been studied, and they will be reported in a separate article. As discussed in Section 2, the changes in the particle diameters will drive changes in the elastic properties of the system. Thus, simulations have been performed with $\sigma^{\prime }/\sigma$ between 0.95 and 1.05. This corresponds to simulations done in [61] and is within the range of values for which the stable tetragonal systems with molecular inclusions are obtained. The Monte Carlo simulations are $10^{7}$ MC cycles long. The MC cycle is understood as a period in which all particles in the system undergo one trial translation (and rotation for molecules). The tests for the volume changes have been set to occur at every $\sqrt{N}$′th trial move of the particles. The first 10% of MC cycles have been treated as the period in which the system reaches the state of thermodynamic equilibrium and, thus, have been discarded from calculations. For every tested value of $\sigma^{\prime }/\sigma$, at least 20 independent simulations have been performed for systems with spherical inclusions [61] and 200 simulations for systems with molecular inclusions. In the latter case, the molecular position and orientation has been randomly generated to better sample the degenerate crystal system.

## 4. Results and Discussion

**Figure 2:**
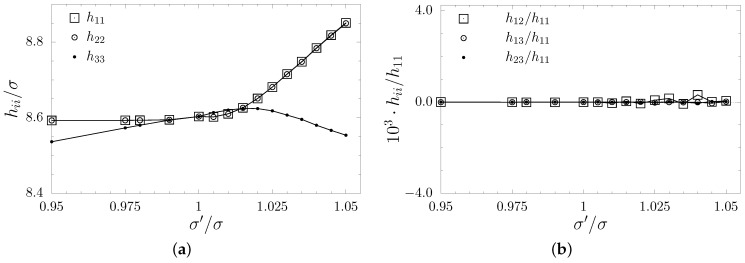
The diagonal elements of the box matrix $h_{ii}$ (**a**) and the ratio of the off-diagonal box matrix elements and $h_{11}$ (**b**) for systems with dumbbell inclusions at $p^{*}=100$, plotted against $\sigma^{\prime }/\sigma$.

**Figure 3:**
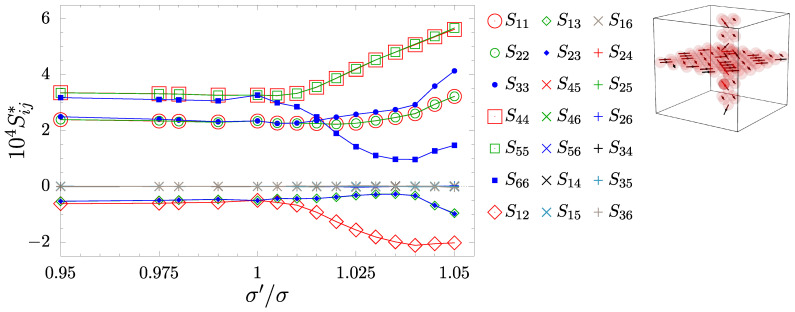
The elements of the elastic compliance matrix $S_{ij}$ for systems with dumbbell inclusions at $p^{*}=100$, plotted against $\sigma^{\prime }/\sigma$.

The inclusion introduced to the f.c.c. crystal, whether composed of spheres or with the disordered phase of diatomic dumbbells, results in a shape change in the sample. In Figure 2, one can observe how the unit supercell changes from cubic ($\sigma^{\prime }/\sigma =1$) to cuboid with a square base. The *x* and *y* dimensions increase to compensate for the increasing atomic diameters in the nanolayer, and the *z* direction shrinks as the remaining particles have more space to compress under pressure. Figure 2b shows the off-diagonal components of the box matrix in relation to $h_{11}$, and it can be seen that they are at least three orders of magnitude less than the diagonal ones. This shows that the supercell has the cuboid shape. Following the change in shape, the symmetry of the system also changes due to the presence of inclusion. In Figure 3, the components of the elastic compliance matrix (S) have been presented and compared with the values of the f.c.c. cubic crystal ($\sigma^{\prime }/\sigma =1$). One can see that lowering the $\sigma^{\prime }/\sigma$ value (decreasing atomic diameters of molecules) does not impact elastic properties significantly. However, when $\sigma^{\prime }/\sigma >1$, the change in crystalline symmetry is clear. It can be noted that the following relations $S_{11}=S_{22}$, $S_{44}=S_{55}$, and $S_{13}=S_{23}$ are fulfilled, and that the symmetry of the crystal with such inclusion is tetragonal (422 symmetry class [80]). Thus, the elastic compliance matrix takes the following form:

(4) $$S=\left[\begin{array}{cccccc} S_{11} & S_{12} & S_{13} & 0 & 0 & 0\\ \cdot & S_{11} & S_{13} & 0 & 0 & 0\\ \cdot & \cdot & S_{33} & 0 & 0 & 0\\ \cdot & \cdot & \cdot & S_{44} & 0 & 0\\ \cdot & \cdot & \cdot & \cdot & S_{44} & 0\\ \cdot & \cdot & \cdot & \cdot & \cdot & S_{66} \end{array}\right].$$

**Figure 4:**
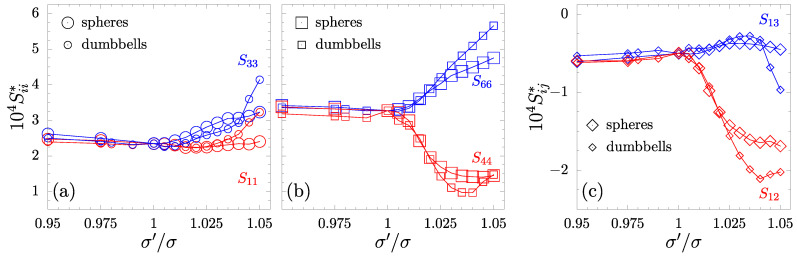
Comparison of elastic compliance matrix elements $S_{ij}$ for systems with inclusions composed of hard spheres [61] (large symbols) and the ones composed of hard dumbbells (small symbols). The colors distinguish elastic compliance matrix elements $S_{11}$ (red) $S_{33}$ (blue) (**a**), $S_{44}$ (red) $S_{66}$ (blue) (**b**), $S_{12}$ (red) $S_{13}$ (blue) (**c**). The respective pairs are equivilent in the cubic system.

The same symmetry has been observed in the case of the same combination of inclusions filled only by spheres [61]. An important remark should be made here, that as a common reference point to both systems with inclusions, the pure f.c.c. crystal of hard spheres with the diameters σ has been selected. One should be aware that the presence of molecular bonds from dumbbell particles at $\sigma^{\prime }/\sigma =1$ (in that case they are called dimers) will also have a certain (small) impact on the elastic properties. This difference is very small compared with the effects discussed in this work (one can analyse the changes in Figure 3 between $\sigma^{\prime }/\sigma =1$ and 0.99), and thus is ignored. In Figure 4, the comparison of the non-zero S matrix elements between atomic and molecular inclusions have been presented. It can be seen that additional constraints in the form of disordered molecular bonds exert more significant changes in the elastic properties than in the case of atomic systems (especially for $S_{44},S_{66}$(b) and $S_{12},S_{13}$(c)).

**Figure 5:**
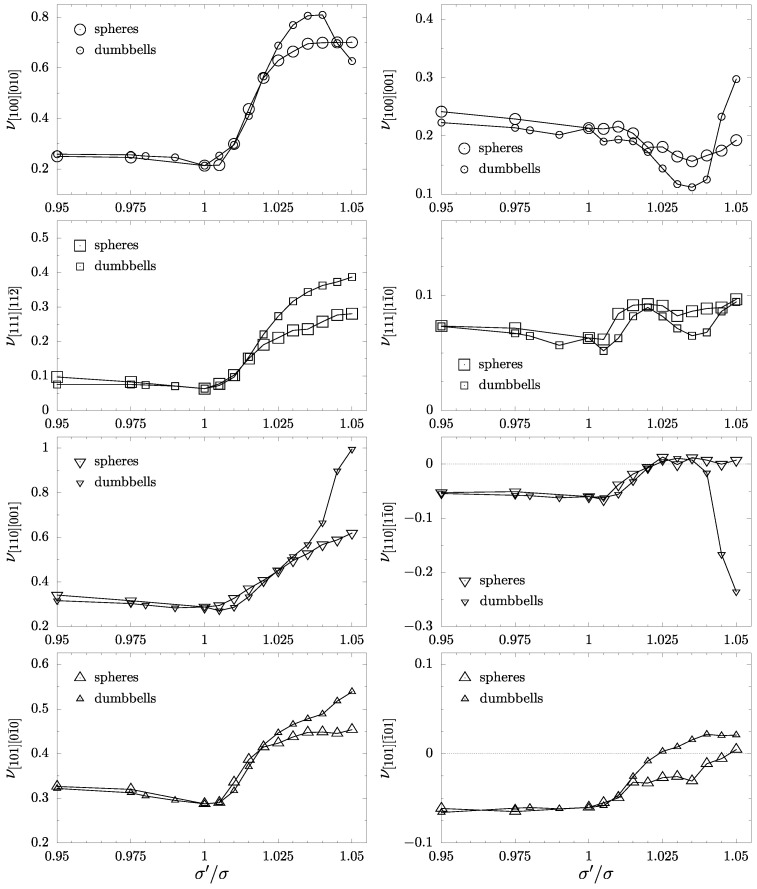
Comparison of the Poisson’s ratio ν in the selected, typical crystallographic directions for systems with inclusions composed of hard spheres [61] (large symbols) and the ones composed of hard dumbbells (small symbols).

**Figure 6:**
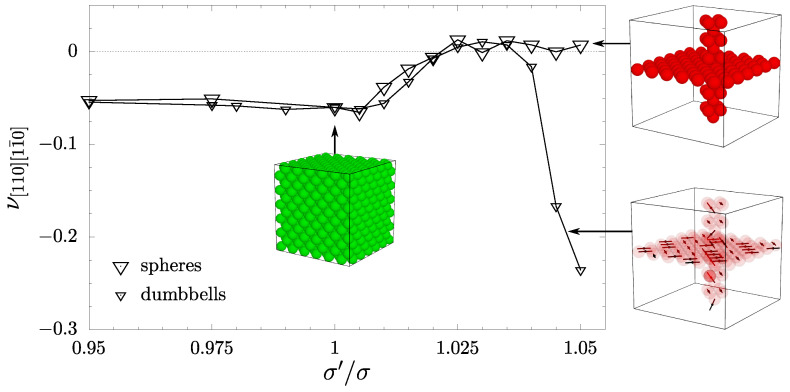
Poisson’s ratio in $\left[110\right]\left[1\bar{1}0\right]$-directions for systems with inclusions composed of hard spheres [61] (large symbols) and the ones composed of hard dumbbells (small symbols). The graphical inserts additionally indicate the type of the system.

The presence of inclusions and the changes in the crystal symmetry are clearly visible in the behavior of the Poisson’s ratio of both systems. In Figure 5 PR in typical crystallographic directions has been presented. The notation of the directions in the form $\nu_{nm}$, where *n* indicates the direction where the load is applied and *m* is the direction in which the Poisson’s ratio is measured. It can be also noted that in the case of cubic symmetry, respective pairs of directions are equivalent. Namely, [100] and [111] are elastically isotropic, so the value of PR in these directions does not depend on *m*. Furthermore, the PR is invariant on the choice of *n* as [110] or [101] in the cubic case. It can be seen in Figure 5 that, in the case of inclusions containing a disordered crystalline phase of hard dumbbells, the PR typically increases more at the given $\sigma^{\prime }/\sigma >1$. The auxetic properties (an inherent property of an f.c.c. crystal) are removed in both, the [110] and [101] directions at s=1.025. Yet, they are still present in the case of spherical inclusions in the latter direction, and they disappear only at $\sigma^{\prime }/\sigma =1.05$. Overall, in the dimer system at $\sigma^{\prime }/\sigma =1.025$, the Poisson’s ratio in some other directions can be found to be negative, but its value is negligibly small. An interesting difference between the spherical and the disordered molecular inclusions is that the latter significantly decreases Poisson’s ratio in the $\left[110\right]\left[1\bar{1}0\right]$ direction when $\sigma^{\prime }/\sigma >1.035$ (see also Figure 6). PR reaches −0.235 and shows a significant decrease over −0.06 for the cubic case.

**Figure 7:**
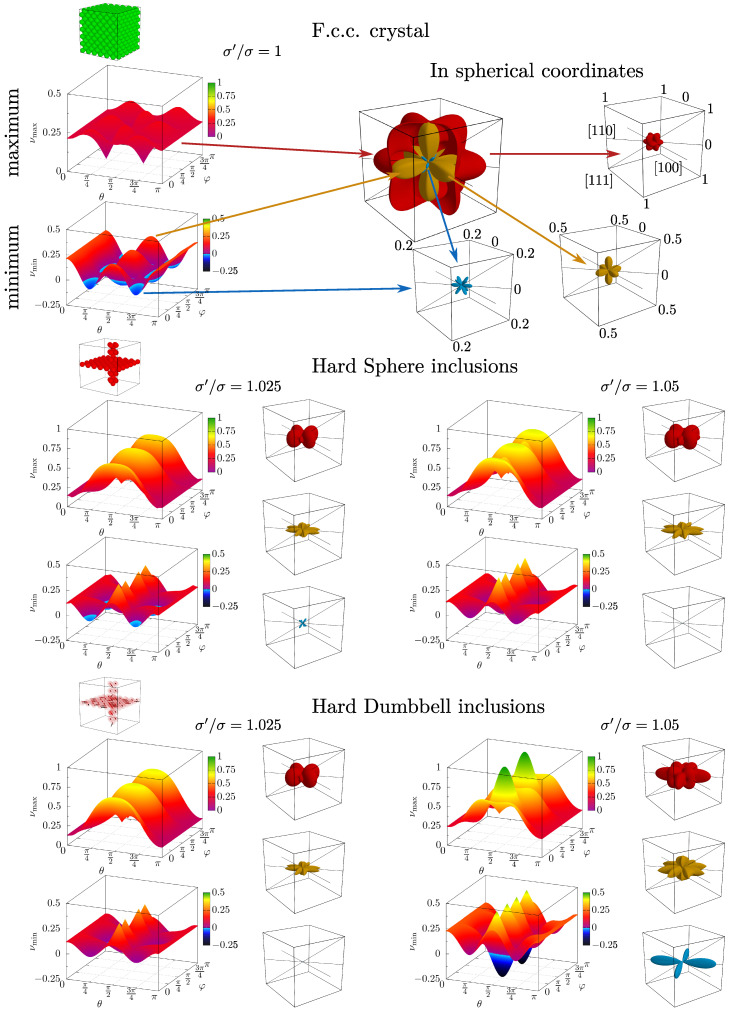
The surfaces of maximum and minimum Poisson’s ratio plotted in the Cartesian coordinates as a function of polar and azimuthal angles (θ,φ) that parametrize the loading direction *n*. The colours on the surfaces indicate the value of the PR defined in the corresponding legend bars. The smaller cubic inserts present the same surfaces plotted in spherical coordinates. Here, the data were divided into maximum PR surface (red) as well as the positive (yellow) and negative (blue) parts of minimum PR surface. The negative part of the maximum PR surface does not exist in this model.

Due to the fact that Poisson’s ratio depends on a pair of mutually orthogonal directions, it is hard to demonstrate all the possible changes that occur to the modifications introduced into the crystalline structure. However, it is possible to show the extreme values of PR in the *m*-directions that correspond to a particular loading direction (*n*). In the Figure 7 the surfaces of the minimum and maximum PR for all the possible loading directions (parametrized by polar and azimuthal angles θ,φ) are presented for the studied systems at selected $\sigma^{\prime }/\sigma$. The presented surfaces limit the possible values of PR in the respective directions shown. It is also convenient to see these surfaces plotted in the spherical coordinates. However, as it can be seen on the lower left part of Figure 7, it is necessary to separately plot the surfaces of the maximum (red) and minimum PR. The latter is additionally separated to positive (yellow) and negative (blue) parts. The corresponding scales apply to all the polar plots presented in the figure. For additional details on the presented plots, see [61]. Looking at the plots, one can easily assess the impact of inclusion itself, as well as the impact of the disordered crystalline phase of dumbbells within the inclusion. One can see that the Poisson’s ratio reaches its extreme values ($\nu_{\max}=1$ and $\nu_{\min}=-0.247$) for the system with molecular inclusions, when $\sigma^{\prime }/\sigma =1.05$. It can also be seen that hard dumbbells introduced into the inclusion cause a significant increase in the overall auxetic properties of the system, when compared with the pure f.c.c. crystal. This is in contrast with the spherical inclusions, which totally removes the auxetic properties from the system.

## 5. Conclusions

The elastic properties of two similar crystal systems of hard particles with structural modifications are compared. The previously studied system of hard spheres containing the combined nanolayer and nanochannel inclusion of hard spheres with different diameters is reviewed and compared with the extended model in which the neighbouring inclusion spheres have been randomly connected in pairs, forming a degenerate crystalline phase of hard dumbbells. It is shown that by introducing the disordered phase into the model system, one can significantly impact its elastic properties. The Monte Carlo simulations in the NpT ensemble showed a significant increase in auxeticity in comparison with the f.c.c. hard sphere crystal. This is also in contrast with the system with nanolayer–nanochannel inclusion composed of unconstrained hard spheres, as in this system, the auxetic properties have been completely removed by the inclusion.

Although the nature of the model interaction used here makes this study purely theoretical, the advances in nanotechnology allow us to fabricate systems even with monoatomic layers [82]. In this context, it is anticipated that ideas presented here will help in designing future real experiments and possibly applications of these effects at a microscale such as sensors or actuators in MEMS systems.

The study presented here focused only on a single size of the inclusion. It is interesting to test the elastic properties of systems with thicker layers and/or wider nanochannels (i.e., different $N_{\mathrm{inc}}/N$ ratios) containing disordered phases. The possibility of out-of-plane orientation of molecules may also have an interesting impact on elastic properties. This will be the subject of future works.

## Data Availability

The raw data supporting the conclusions of this article will be made available by the authors upon request.

## Abbreviations

The following abbreviations are used in this manuscript (listed in order of occurrence in the text):

PR	Poisson’s ratio
HS	Hard Sphere
MC	Monte Carlo
